# Ferulic acid mitigates diabetic cardiomyopathy via modulation of metabolic abnormalities in cardiac tissues of diabetic rats

**DOI:** 10.1111/fcp.12819

**Published:** 2022-08-01

**Authors:** Veronica F. Salau, Ochuko L. Erukainure, Kolawole A. Olofinsan, Nontokozo Z. Msomi, Olayemi K. Ijomone, Md. Shahidul Islam

**Affiliations:** ^1^ Department of Biochemistry University of KwaZulu‐Natal Durban South Africa; ^2^ Department of Pharmacology University of the Free State Bloemfontein South Africa; ^3^ Department of Anatomy University of Medical Sciences Ondo City Nigeria

**Keywords:** cardioprotection, diabetic cardiomyopathy, ferulic acid, lipotoxicity, oxidative stress

## Abstract

Cardiovascular abnormalities have been reported as a major contributor of diabetic mortality. The protective effect of ferulic acid on diabetic cardiomyopathy in fructose‐streptozotocin induced type 2 diabetes (T2D) rat model was elucidated in this study. Type 2 diabetic rats were treated by oral administration of low (150 mg/kg b.w) and high (300 mg/kg b.w) doses of ferulic acid. Metformin was used as the antidiabetic drug. Rats were humanely euthanized after 5 weeks of treatment, and their blood and hearts were collected. Induction of T2D depleted the levels of reduced glutathione, glycogen, and HDL‐cholesterol and the activities of superoxide dismutase, catalase, ENTPDase, and 5′nucleotidase. It simultaneously triggered increase in the levels of malondialdehyde, total cholesterol, triglyceride, LDL‐cholesterol, creatinine kinase‐MB as well as activities of acetylcholinesterase, angiotensin converting enzyme (ACE), ATPase, glucose‐6‐phopsphatase, fructose‐1,6‐bisphophatase, glycogen phosphorylase, and lipase. T2D induction further revealed an obvious degeneration of cardiac muscle morphology. However, treatment with ferulic acid markedly reversed the levels and activities of these biomarkers with concomitant improvement in myocardium structural morphology, which had favorable comparison with the standard drug, metformin. Additionally, T2D induction led to the depletion of 40%, 75%, and 33% of fatty acids, fatty esters, and steroids, respectively, with concomitant generation of eicosenoic acid, gamolenic acid, and vitamin E. Ferulic acid treatment restored eicosanoic acid, 2‐hydroxyethyl ester, with concomitant generation of 6‐octadecenoic acid, (Z)‐, cis‐11‐eicosenoic acid, tridecanedioic acid, octadecanoic acid, 2‐hydroxyethyl ester, ethyl 3‐hydroxytridecanoate, dipalmitin, cholesterol isocaproate, cholest‐5‐ene, 3‐(1‐oxobuthoxy)‐, cholesta‐3,5‐diene. These results suggest the cardioprotective potential of ferulic acid against diabetic cardiomyopathy.

**List of Abbreviations**
ACE
: Angiotensin converting enzyme
AchE
: Acetylcholinesterase
ATPase
: Adenosine triphosphatase
CK‐MB
: Creatine Kinase Myocardial Band
DCM
: Diabetes cardiomyopathy
ENTPDase
: Ectonucleoside triphosphate diphosphohydrolase
GSH
: Reduced glutathione
HDL‐c
: High density lipoprotein‐cholesterol
LDL‐c
: Low density lipoprotein‐cholesterol
MDA
: Malondialdehyde
NFBG
: Nonfasting blood glucose
5’NT
: 5′nucleotidase
SOD
: Superoxide dismutase
T2D
: Type 2 diabetes
**O2** ^**•−**^
: Superoxide anion
ROS
: Reactive oxygen species

## 1 INTRODUCTION

Diabetes is a metabolic disease typified by chronic hyperglycemia resulting from inadequate insulin secretion or impaired insulin action owing to impaired pancreatic β‐cell function or insulin resistance respectively. Both pathophysiologies are typical of type 2 diabetes (T2D), the commonest type of diabetes [1, 2]. It is a metabolic disorder that causes perturbations in the metabolism of biological macromolecules [3]. Diabetes is implicated in increased mortality and morbidity owing to its long‐term complications such as cardiovascular abnormalities [4]. An estimate of 463 million adults were reported to live with diabetes in 2019 with over 4.2 million deaths related to diabetes and its associated complications [2].

Incessant hyperglycemia, insulin resistance, and impaired insulin signaling have been reported as the major risk factors of different diabetic cardiac disorders as seen in the pathogenesis of diabetic cardiomyopathy (DCM). DCM is a condition that results from impaired myocardial structure and function devoid of other cardiac risk factors such as coronary atherosclerosis and hypertension in diabetics [5]. It is characterized by diastolic dysfunction, increased interstitial and perivascular fibrosis, and left ventricular hypertrophy resulting in impaired cardiac morphology and increased risk of heart failure [3].

Oxidative stress, lipotoxicity, dyslipidaemia, and impaired energy metabolism have been reported in the pathogenic mechanism and progression of DCM [3, 6]. Chronic hyperglycemia exacerbates oxidative stress which in turn intensifies insulin resistance that further activate lipolysis, thus leading to accumulation of free fatty acid (FFA). In this state, the mitochondria switches from glucose to fatty acid oxidation as it preferred fuel for ATP production; this increases free radical production which further aggravates oxidative stress and ensuing cardiac cell death [3, 5]. Additionally, increased cardiac angiotensin converting enzymes (ACE) and acetylcholinesterase (AchE) activities have been reported as hallmarks of diabetic heart diseases [7, 8]. Inhibition of these markers is thus have several therapeutic strategies for treating and managing DCM.

Owing to their potent antioxidant and health promoting characteristics, the interest in the use of natural plant products has continued to grow, as they act as therapeutic agents in treating and managing several diseases such as diabetes‐mediated cardiac complications. They also serve as essential ingredient for the development of new drugs in pharmaceutical industries [4, 9]. Among these, natural products are a group of plant secondary metabolites known as the polyphenols whose biological functions have been correlated with their strong antioxidant properties [10].

Ferulic acid (4‐hydroxy‐3‐methoxycinnamic acid) is a common phenolic compound that has been widely studied. It is a derivative of cinnamic acid usually found in ample quantities in fruits, vegetables, grains, and herbs. It exerts its antioxidant effect by acting as singlet oxygen quencher, free radical scavenger, or lipid peroxidation inhibitor [11, 12]. Several studies have reported the pharmacological activities of ferulic acid against many diseases including diabetes [13], neurodegeneration [14, 15], cancers [11], and cardiovascular diseases [16]. Its modulatory effect on lipid profiles and redox status in lowering the risks of cardiovascular diseases has been reported [11]. A study by Chowdhury et al. [17] demonstrated the protective effect of ferulic acid against streptozotocin‐mediated cellular stress in cardiac tissues of diabetic rats. Xu et al. [18] also investigated the effect of the phenolic and its mechanism on cardiomyopathy in diabetic mice. Although studies have demonstrated the effect of ferulic acid on diabetes‐induced cardiac abnormalities, some mechanisms involved in its cardioprotective functions have not been reported.

This study therefore elucidated the effect of ferulic acid on diabetes‐mediated cardiac dysregulated energy metabolism, lipid disturbances, redox imbalance, cardiac tissue morphology, ACE activity as well as cholinergic and purinergic dysfunction in a fructose‐streptozotocin induced rat model of type 2 diabetes (T2D).

## 2 MATERIALS AND METHODS

Ferulic acid (≥99% purity) was purchased from Sigma‐Aldrich, Johannesburg, South Africa.

### 2.1 Ethics statement

This experimental animal research was carried out using the animal ethical clearance (Ethical protocol approval number: AREC/022/019D) approved by the Animal Research Ethics Committee (AREC) of the University of KwaZulu‐Natal, Durban, South Africa.

### 2.2 Animals

Thirty male Sprague–Dawley rats (200–250 g) were collected from Biomedical Research Unit (BRU), University of KwaZulu‐Natal, Westville Campus, Durban, South Africa. The animals were allowed to acclimatize to their environment for 1 week while being provided with standard rat chows and water ad libitum and maintained under standard natural photo period of 12 h light/dark cycle.

### 2.3 Experimental groups and induction of T2D

The rats were randomly divided into six groups made up of five animals each according to the following designation: **HNC** = Normal control (untreated normal rats), **HDC** = Diabetic control (untreated diabetic rats), **HDLF** = Diabetic rats treated with lower dose (150 mg/kg b.w) of ferulic acid, **HDHF** = Diabetic rats treated with high dose (300 mg/kg b.w) of ferulic acid, **HDCM** = Diabetic rats treated with 200 mg/kg b.w of metformin, and **HNTF** = Normal rats treated with high dose (300 mg/kg b.w) of ferulic acid.

T2D was induced in the rats as earlier described by Wilson and Islam [19] after 1 week of acclimatization. Accordingly, 10% fructose solution was supplied to the designated diabetic groups (HDC, HDLF, HDHF, and HDCM) for 2 weeks ad libitum while the normal groups (HNC and HNTF) received ordinary drinking water. At the end of 2 weeks, all the animals were fasted overnight and a one‐time intraperitoneal (i.p) injection of 40 mg/kg b.w streptozotocin (STZ) dissolved in citrate buffer (pH 4.5) was given to animals in the diabetic groups while the animals in normal groups were injected with citrate buffer only. T2D was confirmed exactly a week after STZ injection by measuring their nonfasting blood glucose (NFBG) levels, using a glucometer (GlucoPlus ™). Rats having blood glucose ˃200 mg/dl were regarded as diabetic.

### 2.4 Animal treatment

Beginning from the day of diabetes confirmation, all rats were treated with their respective doses via oral route with the aid of a gastric gavage needle once daily for 5 weeks. The HDLF and HDHF groups, respectively, received low (150 mg/kg b.w) and high (300 mg/kg b.w) doses of ferulic acid. Rats in the HNTF group received high dose (300 mg/kg b.w) of ferulic acid, and the HDCM group (standard drug group) was administered with 200 mg/kg b.w of metformin, while the control groups (HNC and HDC) only received distilled water.

### 2.5 Collection of blood and heart

After 5 weeks treatment period, the rats were humanely euthanized with isofor and whole blood was collected from each rat via cardiac puncture into a 15 ml plain tube. It was centrifuged for 15 min at 3000 rpm (4°C). The resultant serum was collected and stored (−20°C) for subsequent analyses. From each of the animals, the heart tissue was harvested and rinsed in 0.9% NaCl. About 3.5 mm piece of each heart tissue was immediately cut and fixed in labeled tube containing 10% neutral buffered formalin for histological analysis. Afterwards, 0.5 g of each heart tissue was homogenized in 5 ml of 50 mM phosphate buffer containing 1% triton X‐100 (pH 7.5). The homogenates were centrifuged for 10 min (15 000 rpm, 4°C), and each supernatant was stored at −80°C in a labeled 2 ml micro tubes for further analyses.

### 2.6 Determination of cardiac oxidative stress markers

The cardiac oxidative stress biomarkers were analyzed by determining the level of reduced glutathione (GSH) level [20], the activities of catalase [21] and superoxide dismutase (SOD) [22] as well as the level of malondialdehyde (MDA) [23] in the cardiac homogenate.

#### 2.6.1 GSH level

A 300 μl aliquot of the cardiac homogenate was deproteinized with 10% trichloroacetic acid and centrifuged at 3500 rpm for 5 min. In a 96‐well plate, 50 μl of Ellman's reagent (5,5′‐dithiobis‐(2‐nitrobenzoic acid) was added to 200 μl of the supernatant. This was incubated for 5 min on ice before the absorbance was recorded at 415 nm. Cardiac GSH level was extrapolated from a GSH standard curve.

#### 2.6.2 SOD activity

About 170 μl of 0.1 mM diethylenetriaminepentaacetic acid was added to 15 μl of cardiac homogenate in a 96‐well plate. Thereafter, a freshly prepared 15 μl of 1.6 mM of 6‐HD (6‐hydroxydopamine) was added at the point of reading. After gently swirling the plate, the absorbance was read at 492 nm for 3 min at 1 min interval.

#### 2.6.3 Catalase activity

Cardiac catalase activity was determined by incubating 100 μl of cardiac homogenate; 1000 μl of 65 μM hydrogen peroxide in Na_3_PO_4_ buffer (6.0 mM; pH 7.4) at 37°C for 2 min, and absorbance was measured at 240 nm at 1 min interval for 3 min. The reaction was halted by adding 4 ml of ammonium molybdate (32.4 mM) to the mixture.

#### 2.6.4 MDA level

The level of lipid peroxidation was estimated in the cardiac tissues by boiling a reaction mixture made up of 400 μl of tissue homogenate and equal volume of 8.1% sodium dodecyl sulfate (SDS) solution, 1.5 ml of 20% acetic acid, 4 ml of 0.25% thiobarbituric acid (TBA), and 1.7 ml of miliQ water for 1 h. After cooling the mixture, an aliquot of 200 μl was placed into a 96‐well plate and the absorbance was taken at 532 nm. The TBARS concentration was extrapolated from an MDA standard curve to determine the level of cardiac lipid peroxidation.

### 2.7 Determination of cardiac lipase activity

This was determined based on a previously established method [24] with slight modification [25]. Briefly, 100 μl of cardiac homogenate was incubated with 169 μl of 100 mM Tris–HCl in 5 mM CaCl_2_ (pH 7.0) for 15 min at 37°C. Afterwards, 5 μl of p‐nitrophenyl butyrate in dimethyl formamide (10 mM) was included in the mixture and incubated further for 15 min. The absorbance was measured at 405 nm at 1 min interval, and cardiac lipase activity was expressed as the rate of reaction (ΔA/min).

### 2.8 Cardiac lipid profile

The lipid profiles of the cardiac tissues were estimated by measuring total cholesterol, HDL‐cholesterol (HDL‐c), and triglyceride levels in the cardiac homogenate with the aid of an Automated Chemistry Analyzer (Labmax Plenno, Labtest Co. Ltd., Lagoa Santa, Brazil) by using commercially available kits (DiaSys Diagnostic System GmbH, *Holzheim, Germany*) as per manufacture's procedures. The level of LDL‐cholesterol (LDC‐c) was calculated by using Friedewald's equation [26].

### 2.9 Determination of cardiac glucogenic enzymes activities

The cardiac tissue glucogenic enzymes activities were determined by assaying for glucose 6 phosphatase, fructose‐1,6‐bisphosphatase, and glycogen phosphorylase in the cardiac homogenates.

#### 2.9.1 Glucose 6‐phosphatase activity

Glucose 6‐phosphatase activity of cardiac tissue was determined based on the modified method of Balogun and Ashafa [27]. Briefly, 200 μl of cardiac homogenate was added to a mixture of 100 μl of 0.1 M glucose 6‐phosphate, 200 μl of 5 mM KCl, and 1.3 ml of 0.1 M Tris–HCl buffer and incubated at 37°C for 20 min. The reaction was terminated by an addition of 1 ml 10% trichloroacetic acid and allowed to stand on ice for 10 min. Afterwards, the mixture was centrifuged for 10 min at 3000 rpm. About 300 μl aliquot of the supernatant was collected in a 96‐well plate, and the absorbance was measured at 340 nm.

#### 2.9.2 Fructose 1,6 bisphosphatase activity

This was determined based on the previously established protocols [27, 28]. Briefly, 100 μl of cardiac homogenate was mixed with a mixture containing 100 μl of fructose (0.05 M), 1.2 ml of Tris–HCl buffer (0.1 M, pH 7.0), 100 μl of KCl (0.1 M), 250 μl of MgCl_2_ (0.1 M), and 250 μl of EDTA (1 mM) and incubated for 15 min at 37°C. The reaction was halted by adding 10% trichloroacetic acid and centrifuged for 10 min at 3000 rpm (4°C). About 50 μl of 1.25% ammonium molybdate and a freshly prepared 9% ascorbic acid were added to 100 μl of the resulting supernatant and incubated for 20 min at 25°C. The absorbance was read at 680 nm.

#### 2.9.3 Glycogen phosphorylase activity

The cardiac tissues glycogen phosphorylase activity was determined based on the method of [27]. Briefly, 200 μl of the cardiac supernatant was added to 100 μl of 64 mM glucose‐1‐phosphate and 100 μl of 4% glycogen and then incubated for 10 min at 30°C. About 5 ml of 20% ammonium molybdate in concentrated H_2_SO_4_ was used to terminate the reaction. The mixture was further incubated at 30°C for 45 min after adding Elon reducer and distilled water. The absorbance was measured at 340 nm.

### 2.10 Determination of cardiac glycogen content

The glycogen content of cardiac tissue was measured by using a modified protocol of Lo et al. [29]. Saturated 30% potassium hydroxide in sodium sulfate was used to digest 0.5 g of heart tissue and allowed to boil for 30 min. After completely cooling, the mixture was centrifuged for 30 min at 840 g following an addition of 95% ethanol (670 μl). The resulting precipitate was dissolved in distilled water and a 20 μl aliquot added to another 180 μl distilled H_2_O in another tube. About 200 μl of phenol (5%) was mixed with the 200 μl dissolved precipitate (glycogen) or standard. This was followed by a quick addition of 1 ml H_2_SO_4_. The resulting mixture was thoroughly mixed and further boiled for 10 min, and the absorbance was read at 490 nm. The glycogen content was extrapolated from a standard curve.

### 2.11 Determination of cardiac purinergic activities

The cardiac purinergic activities were determined by measuring the activities of adenosine triphosphatase (ATPase) [30, 31], ecto‐nucleoside triphosphate diphosphohydrolase (ENTPDase) [32], and 5′nucleotidase (5′NT) activities [33] in the tissue homogenates.

#### 2.11.1 ATPase activity

A mixture of 200 μl of cardiac homogenate, 1.3 ml of 0.1 M Tris–HCl buffer, 200 μl of 5 mM potassium chloride, and 40 μl of 50 mM adenosine triphosphate (ATP) was incubated for 30 min in shaker set at 37°C. The reaction was halted by adding 1000 μl of distilled H_2_O and ammonium molybdate. Afterwards, a freshly made 9% ascorbic acid was added to the reaction, followed by a 10 min incubation in ice. Absorbance was read at 660 nm.

#### 2.11.2 ENTPDase activity

About 2.0 ml of the ENTPDase buffer made up of 1.5 mM CaCl_2_, 5 mM KCl, 0.1 mM EDTA, 10 mM glucose, 225 mM sucrose, and 45 mM Tris–HCl was mixed with 200 μl of cardiac homogenate and pre‐incubated for 10 min at 37°C. The mixture was then incubated for 20 min in shaker set at 37°C after adding 200 μl of 50 mM ATP. About 2.0 ml of 10% trichloroacetic acid was used to terminate the reaction. A 1.25% ammonium molybdate and a freshly made 9% ascorbate were then added. The reaction was incubated on ice for 10 min, and the absorbance was read at 600 nm.

#### 2.11.3 5′NT activity

A reaction mixture made up of 40 μl of cardiac homogenate, 100 μl of 0.1 M MgCl_2_, and equal volume of 0.1 M Tris–HCl was pre‐incubated for 10 min at 37°C. The mixture was further incubated for 20 min in shaker set at 37°C after an addition of 40 μl of 50 mM ATP. The reaction was halted with 200 μl of 10% trichloroacetic acid and further incubated in ice for 10 min. The absorbance was read at 600 nm.

### 2.12 Determination of cardiac acetylcholinesterase (AchE) activity

The cardiac AchE activity was determined by using Ellman's method [34]. Briefly, 80 μl of cardiac homogenate was added to 40 μl of 3.3 mM Ellman's reagent (pH 7.0) and 200 μl of phosphate buffer (0.1 M, pH 8) and incubated at 25°C for 20 min. About 40 μl of 0.05 M acetylcholine iodide was then added to the mixture, and the absorbance was recorded immediately at 412 nm at 1 min interval for 3 min.

### 2.13 Determination of cardiac ACE activity

ACE activity of the cardiac tissues was determined spectrophotometrically by using a previously established protocol [35] with little modification. Briefly, 40 μl of cardiac homogenate was added to 200 μl of 0.5 mM N‐[3‐(2‐Furyl) acryloyl]‐L‐phenylalanyl‐glycyl‐glycine (FAPGG) and incubated for 10 min. The absorbance was measured at 345 nm at 2 min interval. Cardiac ACE activity was calculated using the expression below:

$$\mathrm{ACE}\;\text{activity}\;\left(\mathrm{\Delta A}/\min\right)=\frac{\left(\mathrm{AI}-\mathrm{AF}\right)\text{Sample}-\left(\mathrm{AI}-\mathrm{AF}\right)\text{Blank}}{\text{Time interval}\left(\min\right)}$$

where AI = initial absorbance and AF = final absorbance.

### 2.14 Serum creatine kinase myocardial band (CK‐MB) level

The concentration of the cardiac marker CK‐MB was estimated in the serum with the aid of an Automated Chemistry Analyzer (Labmax Plenno, Labtest Co. Ltd., Lagoa Santa, Brazil) using commercial assay kit (HUMAN Diagnostics Worldwide, Germany) according to the manufacturer's procedure.

### 2.15 Histopathology examination

The formalin‐fixed heart tissues were processed and fixed on slides. The slides were deparaffinized and stained with hematoxylin and eosin. Images were examined and captured with a digital brightfield microscope (OMAX 40‐2000X 3MP Digital Compound Microscope, USA). Myocardial histological integrity was scored using the following scores: +4 for intact myocardial histology with well delineated fibers running parallelly was scored, +3 for presence of wavy myocardial was scored, +2 for fragmentation of myocardial fibers, and +1 for both presence of wavy fibers and fragmentation of myocardial fibers. Additionally, captured micrographs were imported onto open‐source ImageJ software (NIH, USA) for myocytes nuclei counting using the Image J Cell‐counter.

### 2.16 Extraction of cardiac lipid metabolites

The extraction of cardiac lipid metabolite was done based on a slightly modified method as described previously [36]. Briefly, 3 ml of cold chloroform was used to homogenize 0.3 g of cardiac tissues. It was then incubated for 20 min on ice and centrifuged for 10 min at 15 000 g (4°C). The supernatants were collected and subjected to GC–MS profiling metabolites identification.

### 2.17 GC–MS profiling of extracted lipid metabolites

The lipid metabolites which were extracted for the heart tissue were subjected to GC–MS profiling on an Agilent Technologies 6890 Series GC coupled with (an Agilent) 5973 Mass Selective Detector and driven by Agilent chemstation software. A HP‐5MS capillary column was utilized to separate the metabolites. Injections of 1 μl of the samples were made in splitless mode. Other working parameters were as follows: **Carrier gas**: ultrapure helium, **Flow rate**: 60 ml h^−1^, **Initial oven temperature**: 60°C for 2 min, **Final oven temperature**: 285°C increases at the rate of 5°C min^−1^ with a hold time of 3 min, **Ion source temperature**: 230°C, **Quadrupole temperature**: 150°C, and **Electron ionization mode and electron multiplier voltage:** 70 and 1859 V. The metabolites were identified using an inbuilt NIST mass spectral library.

### 2.18 Pathway analysis

The relevant pathways of the identified cardiac metabolites on the effect of ferulic acid on lipid metabolism in the hearts of diabetic rats were identified using pathways enrichment analysis with the aid of MetaboAnalyst 4.0 online server [37].

### 2.19 Statistical analysis

Results are presented as mean ± SD and were analyzed using one‐way analysis of variance (ANOVA). The Tukey's HSD‐multiple range post hoc test was used in obtaining significant differences between means at *p <* 0.05. The statistical analysis was carried out using the IBM Statistical Package for the Social Sciences (SPSS) for Windows, version 23.0 (IBM Corp., Armonk, NY, USA).

## 3 RESULTS

**FIGURE 1:**
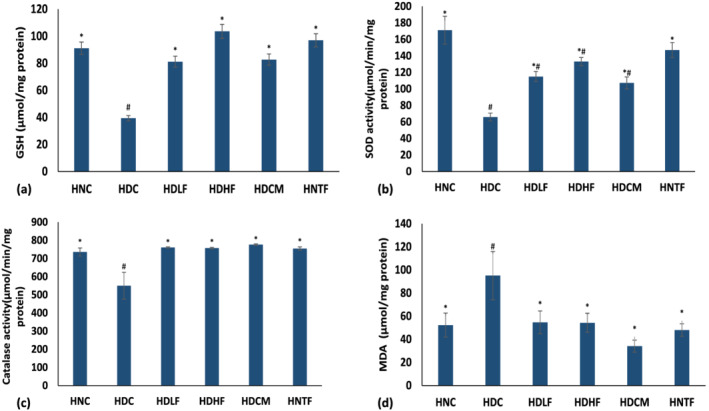
Antioxidant status of experimental groups (a–d). Value = mean ± SD; *n =* 5. *statistically significant (*p <* 0.05) to HDC; #Statistically significant (*p <* 0.05) to HNC. **HNC** = Normal rats, **HDC** = Diabetic control, **HDLF** = Diabetic rats + ferulic acid (150 mg/kg b.w), **HDHF** = Diabetic rats + ferulic acid (300 mg/kg bw), **HDCM** = Diabetic rats + metformin (200 mg/kg b.w), and **HNTF** = Normal rats + ferulic acid (300 mg/kg bw)

As depicted in Figure 1a–d, the level of GSH and the activities of SOD and catalase enzymes in the cardiac tissues were significantly (*p <* 0.05) suppressed after the induction of diabetes; this was also accompanied by a marked increase in MDA level. Treatment with both the low (150 mg/kg b.w) and high (300 mg/kg b.w) doses of ferulic acid restored cardiac GSH levels and activities of SOD and catalase while depleting MDA level. Except for SOD activity, the parameters were restored to near normal levels by ferulic acid and compared favorably with the standard drug metformin.

**FIGURE 2:**
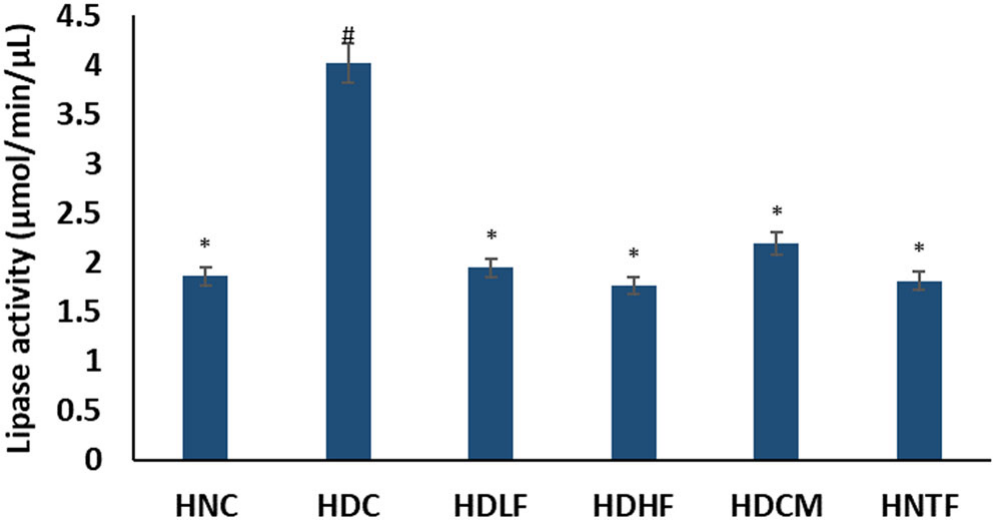
Lipase activity of experimental groups. Value = mean ± SD; *n =* 5. *Statistically significant (*p <* 0.05) to HDC; #Statistically significant (*p <* 0.05) to HNC. **HNC** = Normal rats, **HDC** = Diabetic control, **HDLF** = Diabetic rats + ferulic acid (150 mg/kg b.w), **HDHF** = Diabetic rats + ferulic acid (300 mg/kg bw), **HDCM** = Diabetic rats + metformin (200 mg/kg b.w), and **HNTF**= Normal rats + ferulic acid (300 mg/kg)

As depicted in Figure 2, the lipase activity was markedly elevated in the cardiac tissues after the induction of T2D, treatment with both doses of ferulic acid significantly (*p <* 0.05) reduced lipase activity to levels indistinguishable from the normal control group. Both doses of ferulic acid moderately performed better than metformin. Treatment with high dose of ferulic acid had no effect on the healthy rats (HNTF).

**FIGURE 3:**
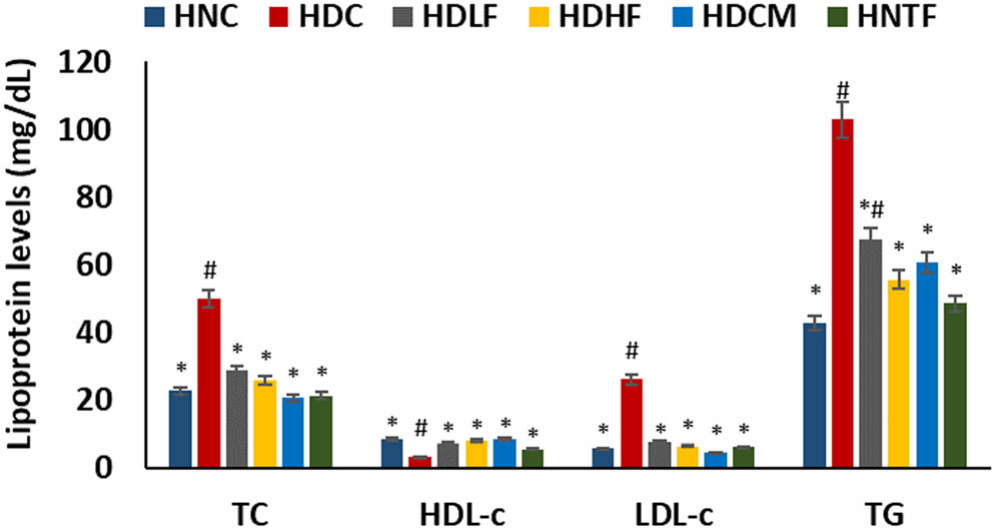
Cardiac lipid profile of experimental groups. Value = mean ± SD; *n =* 5. *Statistically significant (*p <* 0.05) to HDC; #Statistically significant (*p <* 0.05) to HNC. **HNC** = Normal rats, **HDC** = Diabetic control, **HDLF** = Diabetic rats + ferulic acid (150 mg/kg b.w), **HDHF** = Diabetic rats + ferulic acid (300 mg/kg bw), **HDCM** = Diabetic rats + metformin (200 mg/kg b.w), and **HNTF**= Normal rats + ferulic acid (300 mg/kg bw)

As portrayed in Figure 3, induction of T2D altered cardiac lipid profile by significantly (*p <* 0.05) increasing the levels of total cholesterol, triglyceride, and HDL‐cholesterol while depleting LDL‐cholesterol level. The levels were reversed in both the low and high dose ferulic acid‐treated rats and compared with normal and metformin‐treated groups positively. However, the ferulic acid high dose has a notable suppressing effect on triglyceride level compared with the low dose.

As shown in Table 1, induction of T2D led to depletion of the hearts' metabolites as depicted by 40%, 75%, and 33% depletion of fatty acids, fatty esters, and steroids, respectively with concomitant generation of eicosenoic acid, gamolenic acid, and vitamin E. Treatment with the ferulic acid led to the restoration of eicosanoic acid, 2‐hydroxyethyl ester, with concomitant generation of 6‐octadecenoic acid, (Z)‐, cis‐11‐Eicosenoic acid, tridecanedioic acid, octadecanoic acid, 2‐hydroxyethyl ester, ethyl 3‐hydroxytridecanoate, dipalmitin, cholesterol isocaproate, cholest‐5‐ene, 3‐(1‐oxobuthoxy)‐, and cholesta‐3,5‐diene. Treatment with metformin caused the generation of cis‐10‐heptadecenoic acid, undecanedioic acid, cis‐5,8,11,14,17‐eicosapentaenoic acid, octadecanoic acid, 2‐hydroxyethyl ester, and cholesta‐3,5‐diene, while administration to ferulic acid to normal rats led to the generation of octadecanoic acid, 3‐hydroxy‐, methyl ester; triarachine, and cholesta‐3,5‐diene.

**TABLE 1:** GC–MS identified lipid metabolites in heart tissues of experimental groups

Classes	Compounds	HNC	HDC	HDLF	HDHF	HDCM	HNFT
Fatty Acid	Palmitoleic acid	0.54 ± 0.10	0.33 ± 0.04	0.42 ± 0.04	0.54 ± 0.07	0.42 ± 0.05	10.28 ± 1.94
	n‐Hexadecanoic acid	10.59 ± 0.55	12.49 ± 1.04	9.24 ± 1.02	9.81 ± 0.42	10.44 ± 0.55	11.82 ± 0.34
	9,12‐Octadecadienoic acid (Z,Z)‐	10.87 ± 0.98	11.80 ± 1.02	14.27 ± 0.84	8.55 ± 0.47	10.74 ± 0.52	11.20 ± 0.48
	cis‐13‐Eicosenoic acid	9.82 ± 1.27	9.38 ± 0.48	5.09 ± 0.72	ND	3.96 ± 0.02	0.59 ± 0.13
	Octadecanoic acid	8.20 ± 0.27	0.15 ± 0.02	7.48 ± 0.74	7.34 ± 0.91	ND	7.72 ± 0.01
	Arachidonic acid	4.36 ± 0.41	8.32 ± 0.61	5.70 ± 0.63	2.73 ± 0.21	ND	3.53 ± 0.26
	Doconexent	0.25 ± 0.01	ND	ND	ND	ND	ND
	5,8,11,14,17‐Eicosapentaenoic acid	0.34 ± 0.02	ND	ND	ND	ND	ND
	cis‐8,11,14‐Eicosatrienoic acid	0.41 ± 0.15	ND	ND	ND	ND	ND
	2‐Tridecenoic acid, (E)‐	0.32 ± 0.11	ND	ND	ND	ND	ND
	Eicosenoic acid	ND	9.33 ± 1.05	ND	ND	ND	ND
	Gamolenic Acid	ND	0.22 ± 0.02	ND	ND	ND	ND
	6‐Octadecenoic acid, (Z)‐	ND	ND	5.93 ± 1.44	5.98 ± 0.52	ND	ND
	cis‐11‐Eicosenoic acid	ND	ND	ND	3.90 ± 0.13	ND	ND
	Tridecanedioic acid	ND	ND	ND	0.27 ± 0.02	ND	ND
	cis‐10‐Heptadecenoic acid	ND	ND	ND	ND	6.03 ± 0.53	ND
	Undecanedioic acid	ND	ND	ND	ND	0.35 ± 0.06	ND
	cis‐5,8,11,14,17‐Eicosapentaenoic acid	ND	ND	ND	ND	4.84 ± 0.18	ND
Fatty ester	9,12‐Octadecadienoic acid, ethyl ester	1.84 ± 0.25	0.16 ± 0.04	ND	ND	ND	ND
	Eicosanoic acid, 2‐hydroxyethyl ester	0.34 ± 0.02	ND	0.22 ± 0.03	2.89 ± 0.43	ND	0.36 ± 0.01
	5,8,11,14‐Eicosatetraenoic acid, ethyl ester	2.25 ± 0.19	ND	ND	ND	ND	ND
	Methyl 3‐hydroxyhexadecanoate	0.46 ± 0.16	ND	ND	ND	0.44 ± 0.16	ND
	Octadecanoic acid, 2‐hydroxyethyl ester	ND	ND	0.37 ± 0.07	0.35 ± 0.05	0.36 ± 0.05	ND
	Ethyl 3‐hydroxytridecanoate	ND	ND	0.98 ± 0.12	ND	ND	ND
	Octadecanoic acid, 3‐hydroxy‐, methyl ester	ND	ND	ND	ND	ND	0.39 ± 0.13
Fatty alcohol	Dipalmitin	ND	ND	ND	0.22 ± 0.01	ND	ND
	Triarachine	ND	ND	ND	ND	ND	0.50 ± 0.17
Fatty amine	Palmidrol	1.82 ± 0.26	0.55 ± 0.08	0.62 ± 0.06	0.91 ± 0.06	2.81 ± 0.01	1.13 ± 0.05
Steroids	24,25‐Dihydroxyvitamin D	0.27 ± 0.07	ND	ND	ND	ND	ND
	Cholesterol	9.29 ± 3.18	17.14 ± 6.69	19.83 ± 8.50	13.41 ± 5.18	ND	ND
	Squalene	1.88 ± 0.01	1.18 ± 0.02	0.98 ± 0.01	1.21 ± 0.05	1.99 ± 0.24	1.78 ± 0.10
	Vitamin E	ND	0.44 ± 0.19	0.78 ± 0.04	ND	ND	ND
	Cholesterol isocaproate	ND	ND	0.38 ± 0.09	ND	ND	ND
	Cholest‐5‐ene, 3‐(1‐oxobuthoxy)‐	ND	ND	ND	0.60 ± 0.25	ND	ND
	Cholesta‐3,5‐diene	ND	ND	ND	0.44 ± 0.01	0.88 ± 0.12	0.34 ± 0.04

*Note*: Values = mean ± SD; *n =* 3.

Abbreviations: **HDC**, diabetic control; **HDCM**, diabetic rats + metformin (200 mg/kg b.w); **HDHF**, diabetic rats + ferulic acid (300 mg/kg bw); **HDLF**, diabetic rats + ferulic acid (150 mg/kg b.w); **HNC**, normal rats; **HNTF**, normal rats + ferulic acid (300 mg/kg bw).

**FIGURE 4:**
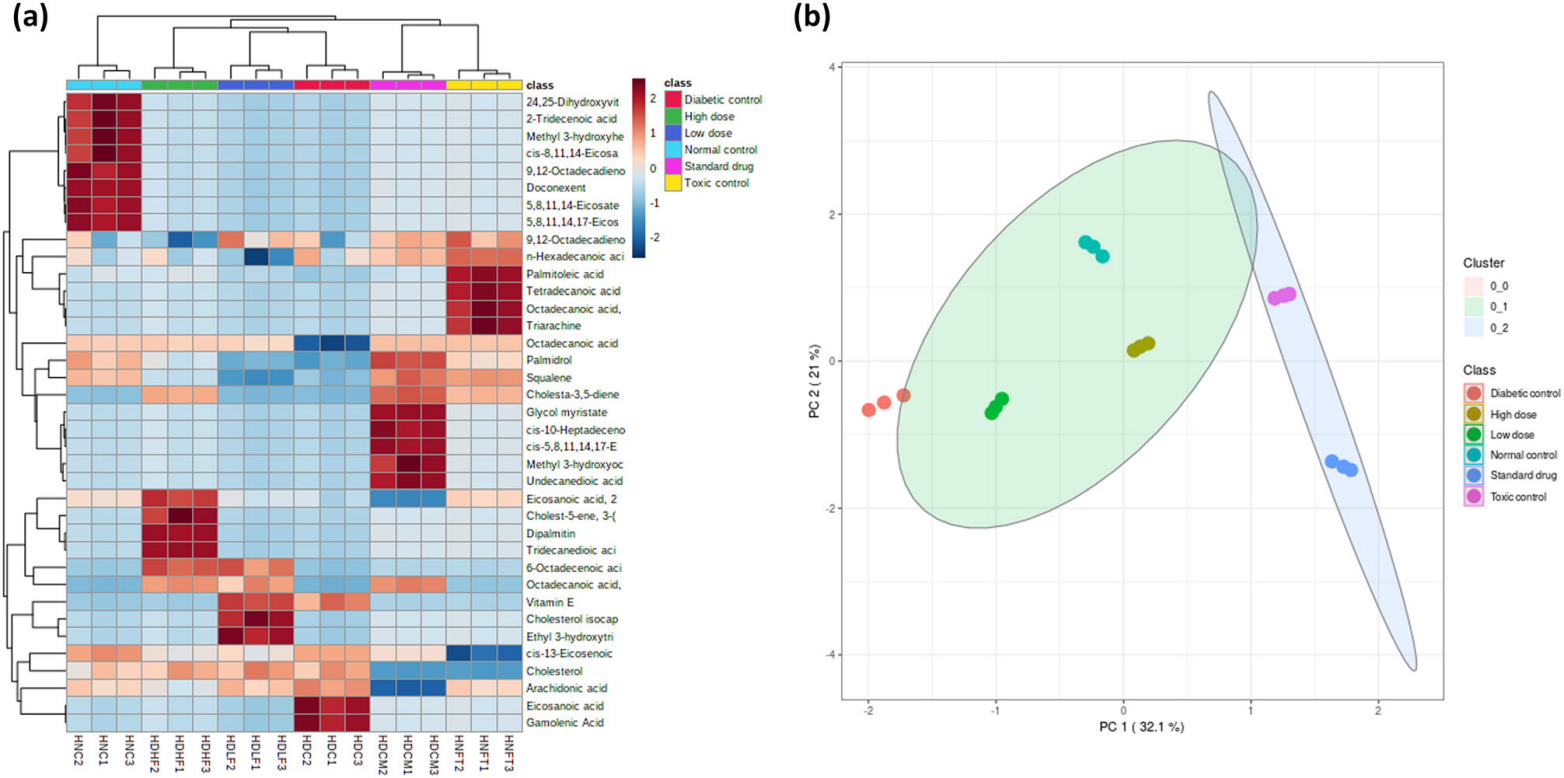
(a) Heat map and (b) score plot between the selected PCs of GC–MS identified lipid metabolites

As shown in Figure 4a, heatmap analysis revealed distinct changes in the cardiac lipid metabolites as well as their distributions across the treatment groups. This is also corroborated by the score plots between the selected PCs which depicts distinct changes and distributions of lipid metabolites across the various treatment groups (Figure 4b), thus depicting a linear distribution of the identified metabolites among the study groups.

**FIGURE 5:**
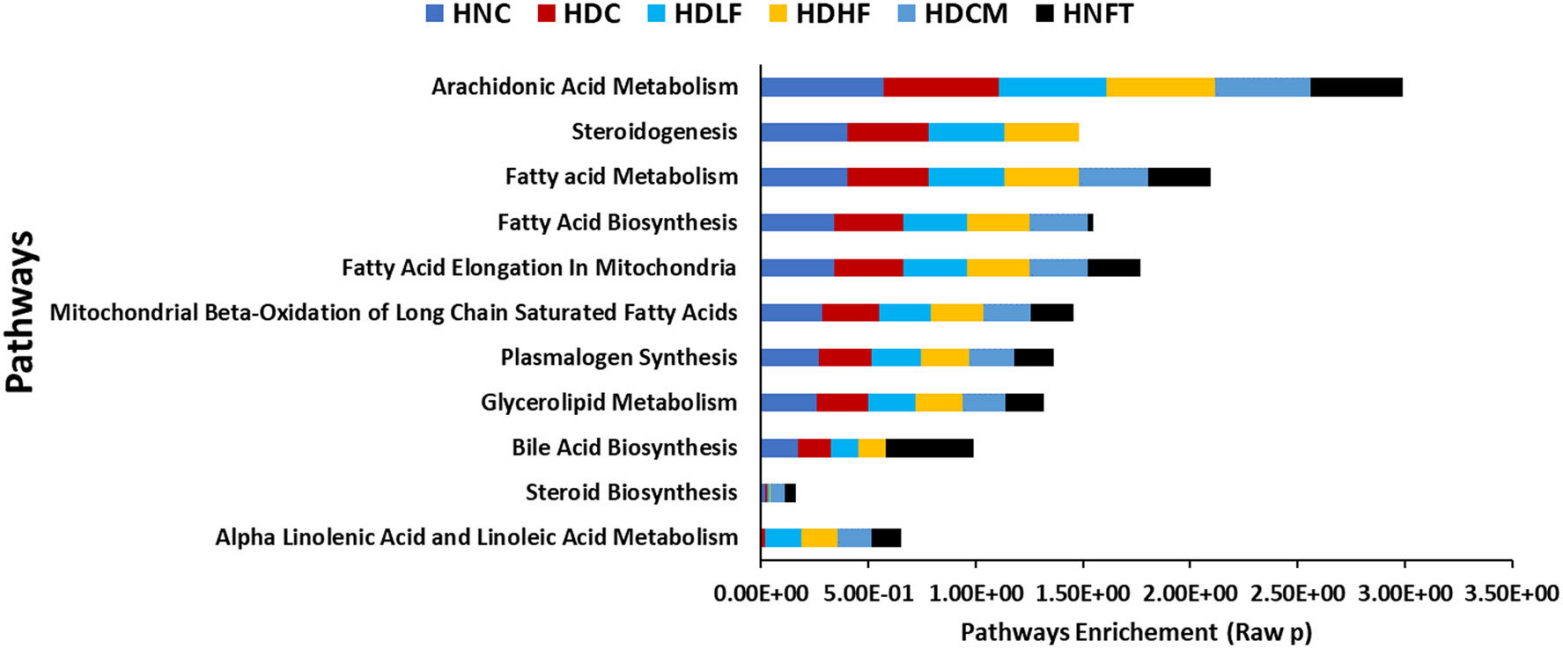
Enrichment ratio of identified pathways in experimental muscle tissues

Pathway enrichment analysis revealed that induction of T2D had no effect on cardiac lipid metabolic pathways as shown in Figure 5. However, treatment with metformin led to the inactivation of bile acid biosynthesis and steroidogenesis pathways, while the administration of ferulic to normal rats led to the inactivation of steroidogenesis.

**FIGURE 6:**
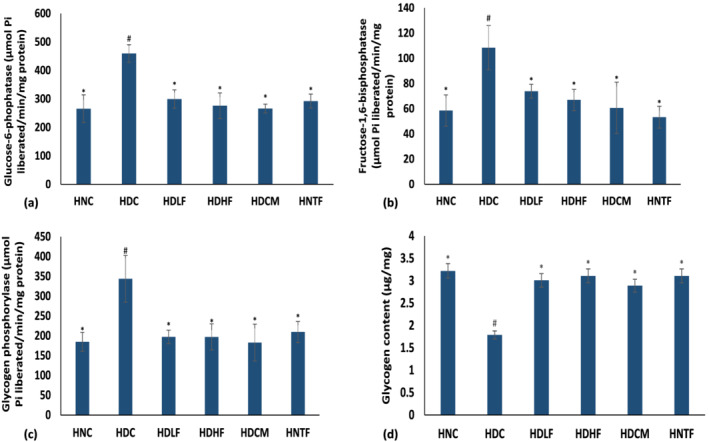
(a) Glucose 6‐phosphatase, (b) fructose‐1,6‐biphosphatase, (c) glycogen phosphorylase, and (d) glycogen content of experimental groups Value = mean ± SD; *n =* 5. *Statistically significant (*p <* 0.05) to HDC; #Statistically significant (*p <* 0.05) to HNC. **HNC** = Normal rats, **HDC** = Diabetic control, **HDLF** = Diabetic rats + ferulic acid (150 mg/kg b.w), **HDHF** = Diabetic rats + ferulic acid (300 mg/kg bw), **HDCM** = Diabetic rats + metformin (200 mg/kg b.w), and **HNTF**= Normal rats + ferulic acid (300 mg/kg bw)

The activities of cardiac glucose‐6‐phosphatase, fructose‐1,6‐bisphosphatase, and glycogen phosphorylase were significantly (*p <* 0.05) increased with notable reduction in glycogen content following T2D induction as portrayed in Figure 6a–d. Like the metformin‐treated group, the glucogenic enzymes activities and glycogen content were reversed in the low and high dose ferulic acid‐treated groups and compared favorably with the normal groups.

**FIGURE 7:**
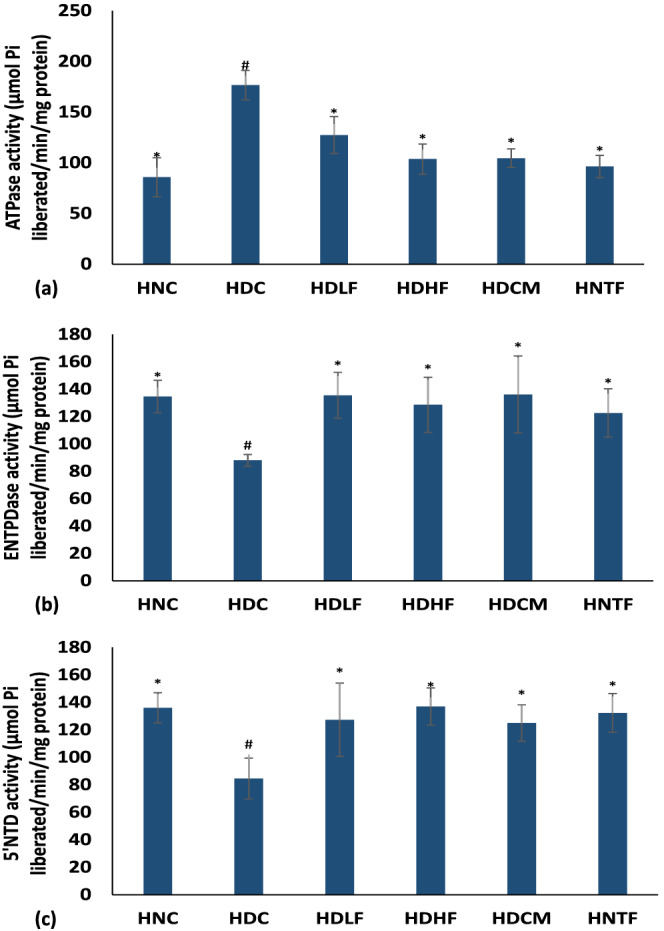
(a) ATPase, (b) E‐NTPDase, and (c) 5′NTD activities of experimental groups. Value = mean ± SD; *n =* 5. *Statistically significant (*p <* 0.05) to HDC; #Statistically significant (*p <* 0.05) to HNC. **HNC** = Normal rats, **HDC** = Diabetic control, **HDLF** = Diabetic rats + ferulic acid (150 mg/kg b.w), **HDHF** = Diabetic rats + ferulic acid (300 mg/kg bw), **HDCM** = Diabetic rats + metformin (200 mg/kg b.w), and **HNTF**= Normal rats + ferulic acid (300 mg/kg bw)

As shown in Figure 7a–c, induction of T2D resulted in significant (*p <* 0.05) increase in ATPase activity and depleted activities of ENTPDase and 5′NT. The activities were reversed to near normal levels on treatment with both doses ferulic acid as does metformin.

**FIGURE 8:**
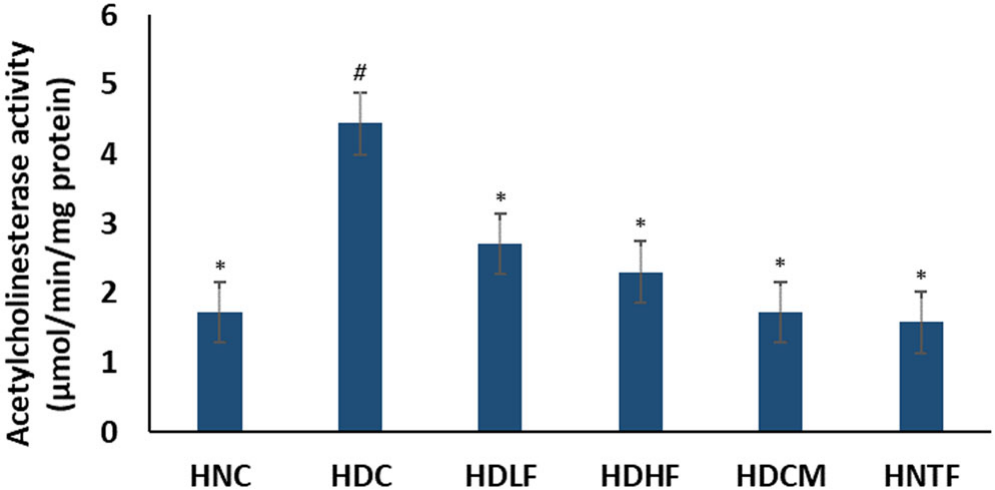
Acetylcholinesterase activity of experimental groups. Value = mean ± SD; *n =* 5. *Statistically significant (*p <* 0.05) to HDC; #Statistically significant (*p <* 0.05) to HNC. **HNC** = Normal rats, **HDC** = Diabetic control, **HDLF** = Diabetic rats + ferulic acid (150 mg/kg b.w), **HDHF** = Diabetic rats + ferulic acid (300 mg/kg bw), **HDCM** = Diabetic rats + metformin (200 mg/kg b.w), and **HNTF**= Normal rats + ferulic acid (300 mg/kg bw)

Cardiac acetylcholinesterase activity which was significantly (*p <* 0.05) elevated on T2D induction was significantly (*p <* 0.05) reduced on treatment with both doses of ferulic acid as indicated in Figure 8. Metformin group (HDCM) showed better activity than ferulic acid‐treated groups. High dose of ferulic acid had no effect on the healthy rats (HNTF).

**FIGURE 9:**
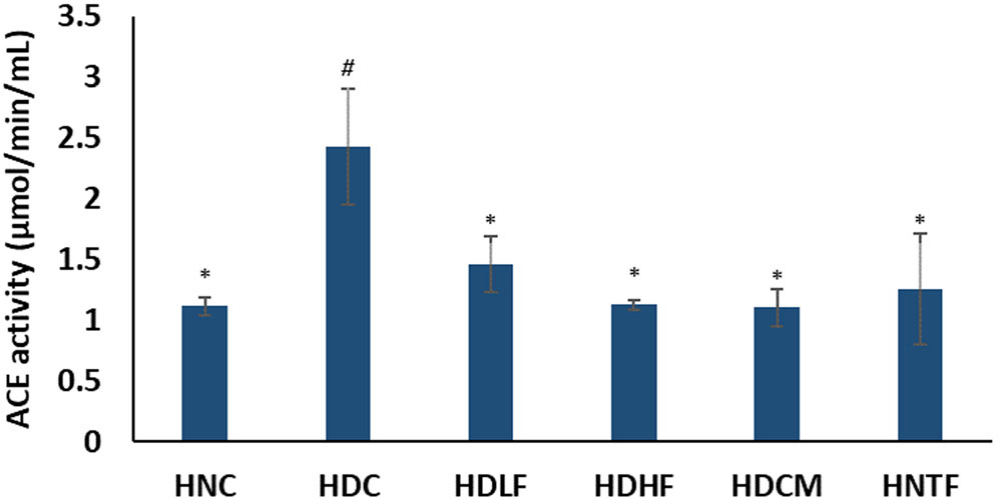
Angiotensin converting enzyme (ACE) activity of experimental groups. Value = mean ± SD; *n =* 5. *Statistically significant (*p <* 0.05) to HDC; #Statistically significant (*p <* 0.05) to HNC. **HNC** = Normal rats, **HDC** = Diabetic control, **HDLF** = Diabetic rats + ferulic acid (150 mg/kg b.w), **HDHF** = Diabetic rats + ferulic acid (300 mg/kg bw), **HDCM** = Diabetic rats + metformin (200 mg/kg b.w), and **HNTF**= Normal rats + ferulic acid (300 mg/kg bw)

As represented in Figure 9, induction of T2D significantly (*p <* 0.05) increased the activity of cardiac ACE. Both doses of ferulic acid significantly (*p <* 0.05) lowered ACE activity. The ferulic acid high dose treated group (HDHF) bear favorable comparison with the normal and standard drug groups.

**FIGURE 10:**
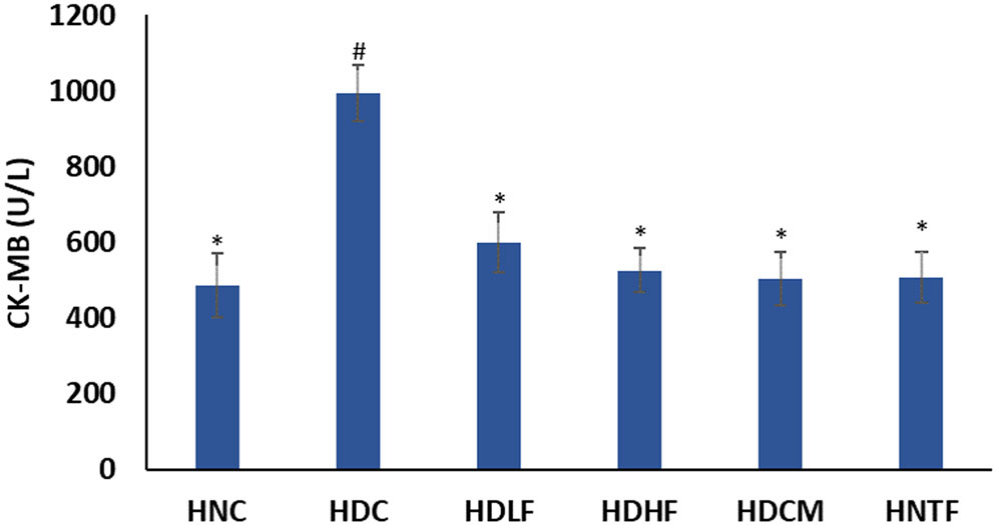
Creatine kinase myocardial band (CK‐MB) level of experimental groups. Value = mean ± SD; *n =* 5. *Statistically significant (*p <* 0.05) to HDC; #Statistically significant (*p <* 0.05) to HNC. **HNC** = Normal rats, **HDC** = Diabetic control, **HDLF** = Diabetic rats + ferulic acid (150 mg/kg b.w), **HDHF** = Diabetic rats + ferulic acid (300 mg/kg bw), **HDCM** = Diabetic rats + metformin (200 mg/kg b.w), and **HNTF**= Normal rats + ferulic acid (300 mg/kg bw)

Serum CK‐MB level of the experimental animals was significantly (*p <* 0.05) elevated on T2D induction as represented in Figure 10. This was significantly (*p <* 0.05) reduced to levels indistinguishable from the normal groups and metformin‐treated rats in both the low and high dose ferulic acid‐treated (HDLF and HDHF) groups.

**FIGURE 11:**
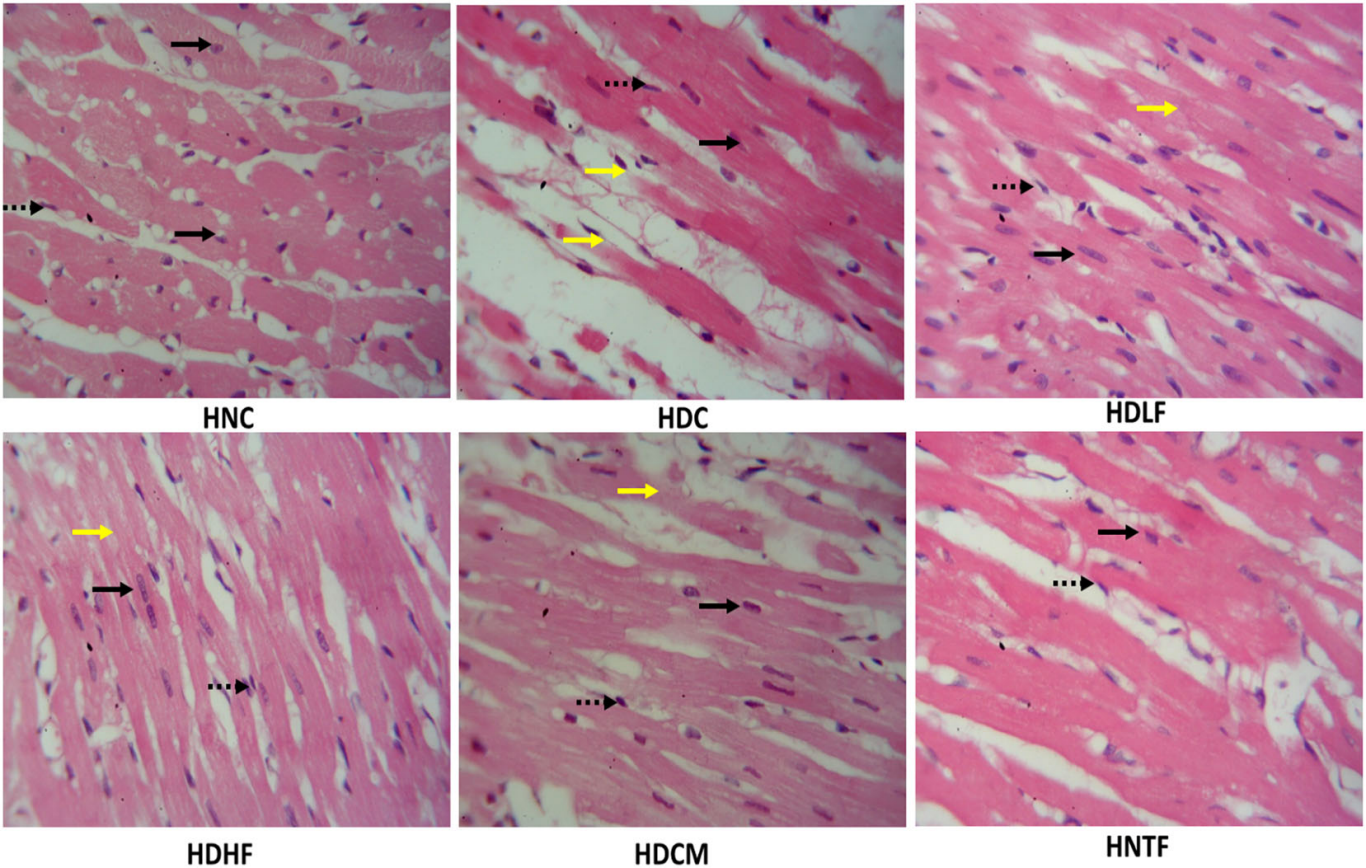
Histological changes in heart of experimental groups. H&E; Magnificatio*n =* x400. Black Arrows – myocyte nuclei; dashed arrows – endothelial nuclei; yellow arrows – myocardial fibers fragmentation. **HNC** = Normal rats, **HDC** = Diabetic control, **HDLF** = Diabetic rats + ferulic acid (150 mg/kg b.w), **HDHF** = Diabetic rats + ferulic acid (300 mg/kg bw), **HDCM** = Diabetic rats + metformin (200 mg/kg b.w), and **HNTF**= Normal rats + ferulic acid (300 mg/kg bw)

As represented in Figure 11, cardiac morphological examination showed that the normal control group (HNC) had normal heart histology with clearly delineated myocytes containing one or two nuclei, as well as endothelial nuclei in the capillary spaces between myocytes. Normal cardiac histology was, however, impaired on induction of T2D, which led to obvious degenerating myocardial fibers presenting as fibers fragmentation. Treatment with both doses of ferulic acid improved the heart histology, with mild to moderate fragmentation of myocardial fibers similar to metformin treatment. High dose of ferulic acid has no effect on cardiac normal (HNTF) morphology.

**FIGURE 12:**
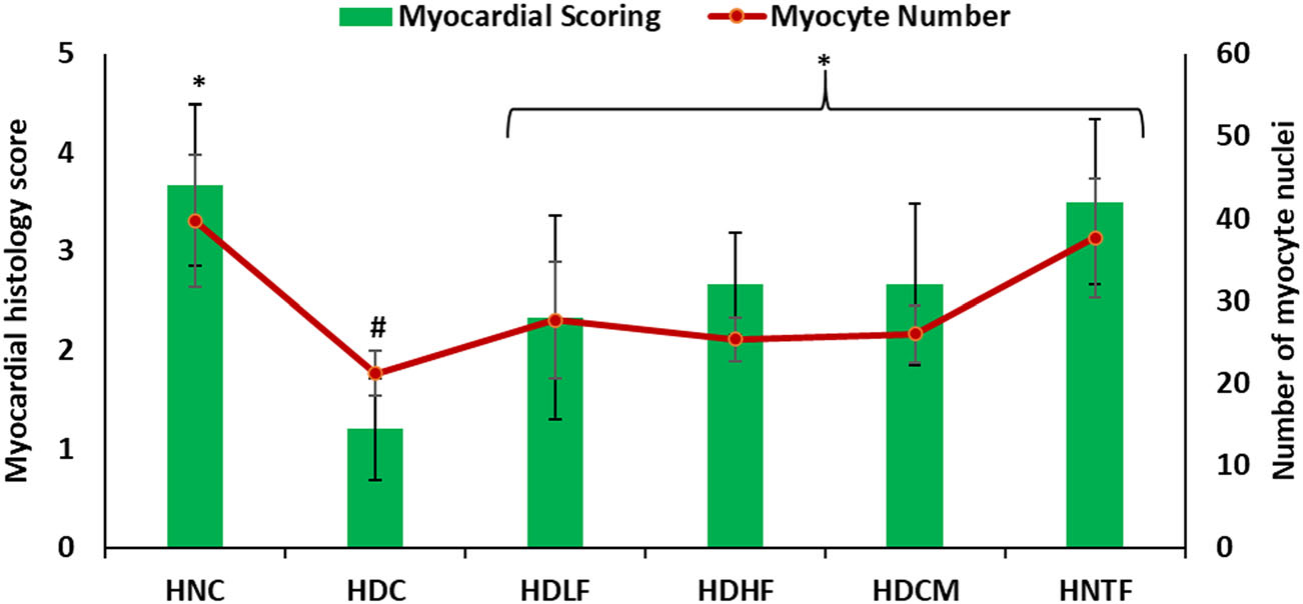
Myocardial score and myocyte numbers of cardiac tissues of experimental groups. Value = mean ± SD; *n =* 5. *Statistically significant (*p <* 0.05) to HDC; #Statistically significant (*p <* 0.05) to HNC. **HNC** = Normal rats, **HDC** = Diabetic control, **HDLF** = Diabetic rats + ferulic acid (150 mg/kg b.w), **HDHF** = Diabetic rats + ferulic acid (300 mg/kg bw), **HDCM** = Diabetic rats + metformin (200 mg/kg b.w), and **HNTF**= Normal rats + ferulic acid (300 mg/kg bw)

Induction of T2D led to significant (*p <* 0.05) decrease in the myocardial scores and myocyte numbers as depicted in Figure 12 These values were significantly increased following treatment with both doses of ferulic acid as well as metformin. Administration of ferulic acid at a high dose to normal rats had no effect of on the myocardial scores and myocyte numbers of the cardiac tissues.

## 4 DISCUSSION

Owing to series of pathogenic mechanisms involved in diabetes, namely, chronic systemic hyperglycemia, oxidative stress, dyslipidaemia, lipotoxicity, and altered energy metabolism, the disease predisposes patients to several vascular complications such as DCM and high mortality rate [3, 4]. Most diabetes treatment strategies and regimens are targeted towards controlling these pathogenic factors which in turn circumvent the development and progression of its associated vascular complications. Plant natural products which are known strong antioxidant have come in handy in managing many diseases due to their several reported therapeutic activities. In the present study, we reported the mitigating effect of ferulic acid on metabolic abnormalities linked to cardiomyopathy in T2D rats.

Oxidative stress plays a significant role in the onset of diabetes and development of DCM [38]. In diabetes state, chronic hyperglycemia exacerbated by insulin resistance induces overproduction of reactive oxygen species (ROS) and causes oxidative damage to multiple organs [39]. Hyperglycemia mediated‐ROS production and cellular membrane lipid peroxidation concomitantly suppress cardiac endogenous antioxidant systems such as reduced GSH, SOD, and catalase and exacerbate myocardial damage [17, 40]. In the present study, the depleted cardiac GSH level, SOD, and catalase activities as well as increased MDA level observed in the untreated diabetic (HDC) group (Figure 1) insinuate a weakened cardiac antioxidant system, onset of lipid peroxidation, and oxidative stress in T2D. Antioxidant therapy is essential in circumventing or delaying the development of diabetic cardiovascular abnormalities [40]. The increased GSH level, activities of SOD and catalase, and reduced MDA content after treatment with ferulic acid suggest an improvement in cardiac antioxidant capacity. This insinuates the antioxidant therapeutic effect of ferulic acid against cardiac oxidative damage and agrees with previous reports [17].

The mammalian heart requires an uninterrupted supply of ATP as energy for optimum contractile performance [41]. Thereby, the heart maintains optimum metabolic flexibility by a swift switch to a preferred substrate, namely, FFA, carbohydrates (glucose and lactate), amino acids, and ketones, to oblige different physiological and pathophysiological conditions depending on energy demand [41, 42]. However, altered regulation of substrate metabolism has been implicated in DCM [43]. Chronic hyperglycemia, insulin resistance, or insulin deficit result in excess generation of FFA from adipose tissue to the heart and increase cardiac lipase activity. Accumulation of FFA thus inhibits myocardial glucose uptake, leading to cardiac preferential shift to FFA utilization over glucose for energy generation [44, 45]. The high activity of cardiac lipase (Figure 2) in the untreated diabetes group (HDC) insinuates an accumulation of FFA and a metabolic shift in substrate oxidation. The result indicates lipotoxicity, as it has been reported that FFA overload in the heart further increases accumulation of FFA intermediates and triglycerides which results in ROS generation and subsequent cardiac lipotoxicity and cardiac damage [44, 46]. Thus, the elevated cardiac triglyceride, cholesterol, and LDL‐c levels of the untreated diabetic group (Figure 3) may be attributed to the elevated lipase activity (Figure 2). This is further corroborated by the altered cardiac lipid metabolites in the untreated diabetic groups (Table 1 and Figure 4). The decreased activity of lipase (Figure 2) following ferulic acid treatment indicates a reduction in FFA level as depicted by the depletion of the diabetic‐generated lipid metabolite (Table 1) with concomitant depleted cardiac triglyceride, cholesterol, and LDL‐c as well as elevated HDL‐c levels, which also insinuates decreased cardiac lipotoxicity [3]. These results corroborate previous reports on altered lipid metabolism in DCM in experimental diabetes [3]. These observations thus suggest an improvement in cardiac lipid metabolism in DCM. The altered pathways in normal rats administered with ferulic acid (Figure 5) may indicate a toxic effect of the phenolic on cardiac lipid metabolism.

Increased activities of glucose‐6‐phosphatase, fructose‐1,6‐bisphosphatase, and glycogen phosphorylase (Figure 6a–c) as indicated by depleted glycogen content (Figure 4d) in the HDC group suggest an activation of gluconeogenesis and an arrest of glycolysis thus leading to accumulation of intracellular glucose. Long‐lasting accumulation of unused glucose leads to generation of ROS and increased glycation, resulting in glucotoxicity [46]. Myocardial glucotoxicity has been implicated in cardiomyopathy and cardiac damage [47]. However, the use of glucose‐lowering agents is part of the therapeutic strategies employed in abating DCM. The reversal of glucogenic enzyme activities as well as an increased glycogen content (Figure 6a–d) following ferulic acid treatment insinuate an inhibition of gluconeogenesis and activation of glycolysis, thus decreasing glucotoxicity. This corroborates previous reports on the ability of plant natural products in suppressing exacerbated glucogenic activities in cardiotoxicity [45].

The role of adenosine and ATP is critical in sustaining cardiac energy metabolism. Adenosine is produced from the catalytic hydrolysis of ATP and adenosine monophosphate (AMP) by purinergic enzymes [3, 48]. ATP is critical for myocardial pump function and cardiomyocyte viability [49], while adenosine has been reported for its activities in coronary vasoregulation and as a powerful dilator [50]. Therefore, alteration in their concentrations may lead to altered cardiac performance and heart failure [51]. The elevated activities of ATPase with concomitant depleted activities of ENTPDase and 5′NT following T2D induction (Figure 7a–c) indicate a reduction in cardiac ATP and adenosine levels. This suggests a weakened cardiac energy reservoir and a reduction in cardiac performance. Adequate availability of cardiac ATP and adenosines is critical for cardiac functions [46]. The reversal in the activities of purinergic enzymes in the ferulic acid‐treated groups (HDLF and HDHF) therefore indicates restoration of ATP and adenosine concentrations in cardiac tissues. This suggests an improvement in cardiac pumping functions and a cardioprotective effect of ferulic acid against DCM.

Cardiac acetylcholine which can be hydrolyzed to acetate and choline by acetylcholinesterase (AchE) plays critical functions in regulating changes in cardiac rate and contractility and plays protective roles in cardiac diseases. A depleted level of acetylcholine thus alters cardiac activities [52]. Impairment in cholinergic activities as typified by elevated activities of AchE has been reported in DCM [3] as this leads to depleted cardiac acetylcholine level. The increased activity of cardiac AchE in the untreated diabetic (HDC) group (Figure 8) indicates altered cardiac cholinergic function owing to suppressed cardiac acetylcholine levels. This corroborates previous reports on increased AchE activity in diabetic hearts [3]. Reports have indicated the enhancement of cholinergic transmissions and improvement of cardiac functions in cardiovascular diseases with the aid of acetylcholinesterase inhibitors [53, 54]. Ferulic acid portrayed an acetylcholinesterase inhibitory property by its ability to deplete the activity of cardiac acetylcholinesterase in the ferulic acid‐treated experimental groups (HDLF and HDHF).

The elevated activity of ACE in the untreated diabetic group (Figure 9) indicates an elevated cardiac level of angiotensin II. ACE is involved in the catalytic hydrolysis of angiotensin I to Angiotensin II, a bioactive octapeptide that facilitates vasoconstriction and a key player in pathophysiology and progression of diabetic micro and macrovascular complications, including DCM [3, 55, 56]. Increased expression of ACE has thus been indicated in cardiac disorders [55]. However, ACE inhibitors have been reported as beneficial targets in therapeutic manipulations of cardiopathies and cardiotoxicity [57, 58]. The reduced cardiac ACE activity after treatment with ferulic acid thus indicates depleted angiotensin II level. This suggests the inhibitory effect of ferulic acid on cardiac ACE and its cardioprotective effect against DCM.

High serum levels of CK‐MB is a powerful indictor of risk of cardiovascular complications [59]. Cardiac leakage of CK‐MB into the serum has been associated with DCM [60]. Elevated serum level of CK‐MB in the untreated diabetic group (Figure 10) is an indication of DCM, and this corroborates previous report on increased serum CK‐MB level in myocardial hypertrophy [61]. The marked reduction in serum CK‐MB concentration in the ferulic acid‐treated groups indicates its cardioprotective effect, and this observation agrees with previous report [60].

The degeneration of muscle fibers in cardiac tissues of the untreated diabetic group (Figure 11) as indicated by degenerate and fragmented vacuolated myocytes depicts an alteration in myocardium morphology. With hyperglycemia‐induced oxidative stress playing a pivotal role, altered myocardial structure and cell death are major hallmarks of DCM and has been implicated in increased risk of heart failure [4, 5]. The delineated myocyte with intact myocytic nuclei and endothelial nuclei presented in the cardiac tissues of ferulic acid‐treated groups is an indication of improved myocardial structure and its ability to protect against DCM. This is further corroborated by the elevated myocardial score and myocyte numbers in ferulic acid‐treated groups (Figure 12).

## 5 CONCLUSION

It can be concluded from the present study that ferulic acid demonstrated cardioprotection against cardiac oxidative damage and DCM by mitigating oxidative stress, lipid accumulation, and purinergic dysfunction, improving energy metabolism, and structural morphology while inhibiting ACE and acetylcholinesterase activities in cardiac tissues of T2D rats. Thus, the study provided broader understanding on the mechanisms by which the phenolic executes its protective effect in diabetic‐induced cardiac complications. Further molecular studies may be considered to further fathom these cardioprotective mechanisms of ferulic acid.

## CONFLICT OF INTEREST

The authors report no conflict of interest relating to this work.

## ETHICS STATEMENT

This experimental animal research was carried out using the animal ethical clearance (Ethical protocol approval number: AREC/022/019D) approved by the Animal Research Ethics Committee (AREC) of the University of KwaZulu‐Natal, Durban, South Africa.

## AUTHOR CONTRIBUTIONS

Conceptualization: MSI and VFS. Experimental design and analyses: OLE, KAO, NZM, and OKI. Original draft: VFS, OLE, and OKI. Revision and approval of manuscript: all authors. Supervision: MSI.

## PERMISSION TO REPRODUCE MATERIAL FROM OTHER SOURCES

Not applicable.

## Data Availability

All data generated from this study are presented in the article, and no data from third party have been in this article.
